# Inguinoscrotal Hernia, a Possible Cause of Rapidly Developing Fetal Scrotal Mass: Case Report and Literature Update

**DOI:** 10.3390/healthcare12050583

**Published:** 2024-03-02

**Authors:** Ramona Montironi, Stefano Raffaele Giannubilo, Irene Cappanera, Giovanna Irene Battistoni, Romina Mancinelli, Andrea Ciavattini

**Affiliations:** Clinical Sciences Department, Obstetrics and Gynecology Section, Università Politecnica delle Marche, Via Filippo Corridoni 11, 60123 Ancona, Italy; ramona.montironi@ospedaliriuniti.marche.it (R.M.); irene.cappanera@gmail.com (I.C.); giovannairene.battistoni@ospedaliriuniti.marche.it (G.I.B.); romina.mancinelli@ospedaliriuniti.marche.it (R.M.); a.ciavattini@univpm.it (A.C.)

**Keywords:** fetus, inguinoscrotal, hernia, scrotal mass, ultrasound

## Abstract

Inguinoscrotal hernia is a common pediatric disease but a rare condition in the fetus. We present a case, from our institution, of fetal inguinoscrotal hernia with possible rapid development. In addition to our case, we present a literature update on fetal inguinoscrotal hernia in order to enhance the ability to recognize it from the other scrotal masses on ultrasound. Antenatal management, differential diagnosis and postnatal management are also discussed.

## 1. Introduction

Inguinal hernia is common among neonates and infants, as it occurs in up to 4% of full-term babies and up to 9–11% of those born prematurely due to the persistence of a patent processus vaginalis (PPV) [1,2]. It occurs more frequently in males (90%) and represents one of the main indications for pediatric surgery since a timely correction is recommended to reduce the risk of bowel incarceration, strangulation and overall morbidity and mortality [1]. Surgery, called herniorrhaphy, consists of the incision of the groin and the repositioning of the hernia sac in the abdomen. However, the inguinoscrotal hernia is rarely diagnosed in utero, and, thus, it is poorly considered in the differential diagnosis of fetal scrotal masses; little is known about its prenatal pathogenesis, presentation and management.

We present a case report and a literature update of fetal inguinoscrotal hernia to characterize its antenatal sonographic features and management. 

## 2. Case Report

**Figure 1:**
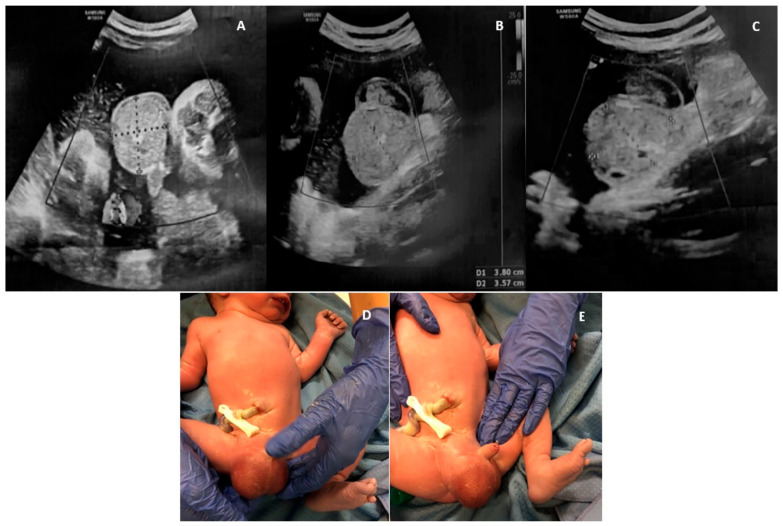
A case of fetal inguinoscrotal hernia (fetal sonographic findings at 38 + 6 weeks of gestation: (**A**,**B**) a 4.0 × 3.5 cm right scrotal mass, mainly solid, with regular wall, complex echotexture, no blood flow signal and (**C**) a few small echo-free cystic areas in its interior; (**D**,**E**) postnatal examination).

A 39-year-old woman, gravida 3, para 1, was referred to our institution at 38 + 6 gestational weeks (GW) to perform a caesarean section (CS) for breech presentation, since she had refused the external cephalic version. The pregnancy was complicated by HIV infection and treated with antiretroviral therapy (ART) (Emtricitabina, Tenofovir and Raltegravir) and antidepressant drugs (Citalopram) from the first trimester of pregnancy. Regarding the fetus, routine obstetric scans at 12, 20 and 30 GW did not reveal any abnormalities. At 36 GW, during a routine obstetric visit, a fetal ultrasound (US) showed a mild unilateral pyelectasis and amniotic fluid at the upper limits (deepest pocket of 7.5 cm), with no other anomalies of the fetus. At the time of hospitalization, a routine US was performed to assess the fetal presentation before the CS. During the examination, a 4.0 × 3.5 cm mass was incidentally noted in the right scrotum. The mass was mainly solid, with regular wall, complex echotexture and a few small echo-free cystic areas in its interior. There was no identifiable blood flow signal during the Color Doppler (CD) assessment, as well as no peristaltic movements in the mass and no evidence of intraabdominal bowel dilatation. The homolateral testis was not detected. The contralateral scrotum showed the presence of mild hydrocele. Breech presentation and mild unilateral pyelectasis (anterior to posterior diameter of the renal pelvis, 9 mm) were confirmed, while moderate polyhydramnios was noticed (deepest pocket of 12 cm). Scrotal tumor versus hemorrhage after testicular torsion was questioned; however, the possible rapid onset of the lesion favored the second hypothesis. The day after, an elective CS was conducted at 39 + 0 GW without complications. A male infant weighing 2890 g was delivered with Apgar scores of 9 and 10 at 1 min at 5 min, respectively. Postnatal examination revealed a large, right-sided inguinoscrotal hernia, reducible but readily recurrent (Figure 1). The diagnosis was confirmed by the US of the scrotum, which showed a right inguinal–scrotal hernia with engagement of bowel loops without signs of loop distress. The right testis and epididymis appeared to be abutted to the scrotal floor and showed preserved echotexture. During CD examination, a regular vascular signal was confirmed in the right testis. Surgical correction of the inguinal–scrotal hernia was performed at 15 days of age because of intestinal obstruction since the hernia was no longer reducible. Surgery consisted of a transverse incision of the groin, the individuation of the spermatic cord, and the repositioning of the hernia sac in the abdomen after its incision and verification of the intestinal content. There were no signs of bowel strangulation, but incarceration was confirmed.

## 3. Methods

**Figure 2:**
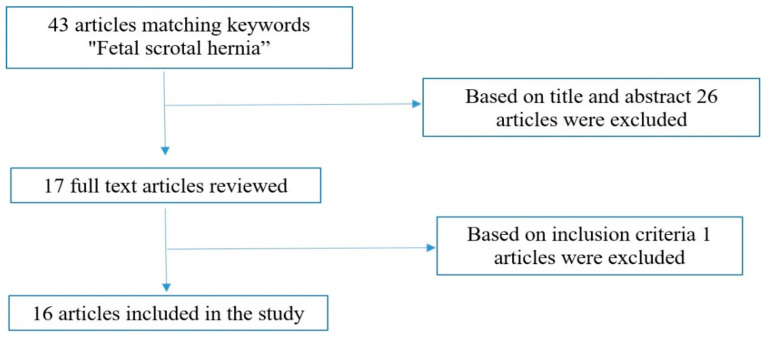
Research strategy flowchart.

We conducted a close literature examination of scientific articles using the PubMed/MEDLINE medical database. The search terms used were “fetal scrotal hernia” from inception to 31 September 2023. Inclusion criteria were all human cases of fetal inguinoscrotal hernia with prenatal US assessment and confirmed by postnatal examination and surgery. Exclusion criteria included all animal cases of fetal inguinoscrotal hernia, cases with no full text available, cases without prenatal US assessment, cases without postnatal confirmation and cases of postnatal inguinoscrotal hernia. A total of 43 articles matched the keywords “fetal scrotal hernia”. The titles and abstracts of these articles were screened by the authors (R.M. and I.C.) to determine which articles could undergo full-text review. A total of 26 articles were excluded based on the title and abstract, and 1 article was also excluded because the full text was not available. A total of 16 papers were finally identified independently and met the inclusion criteria after full text evaluation (Figure 2).

## 4. Results

According to the reported research strategy, we found a total of 18 cases of fetal inguinoscrotal hernia. All the cases are from case reports and are detailed in Table 1. Antenatal diagnosis/suspicion of fetal inguinoscrotal hernia was essentially made via US between 21–39 GW, with a mean of 33 GW, because of the presence of a scrotal mass. Magnetic resonance imaging (MRI) was performed in only 3 cases, revealing the bowel as the location of the lesion. The right side was affected in 12/18 cases. In all the cases (18/18), inguinoscrotal hernia presented at the US as a fetal scrotal mass, whose dimensions ranged between 2.9 × 1.7 cm of the smallest and 6.5 × 5.6 × 6.4 cm of the largest, with the following US features: mainly solid mass (5/18), with complex/mixed/non-homogenous/heterogeneous (9/18) or echogenic/hyperechogenic echotexture (5/18) and few small echo-free cystic areas in its interior (6/18); in the remaining cases, the US findings were not described. From the sagittal view of the fetus, in 1 case, herniation of the bowel through the abdominal wall into the scrotum was noticed. The homolateral testis was found to be laterally displaced in 1 case, not identified in 5/18 cases and not described in 12/18 cases. The contralateral testis was peripherally displaced in 4/18 cases, not mentioned in 13/18 cases and normal in 1/18 cases. In 1 case, a contralateral hydrocele was found. Bowel peristalsis was noticed in 16/18 cases, mainly from the first examination. In 2/18 cases, peristalsis was not observed. Blood flow signal during a CD examination was present in 7/18 cases, absent in 3/18 cases and not described in 8/18 cases. Polyhydramnios was identified in 4/18 cases—3 of them in the context of multiple fetal abnormalities. Intraabdominal bowel dilatation was absent in 8/18 cases, not mentioned in 6/18 cases and present in 4/18 cases; among the latter, 1 was a case of cystic fibrosis (CF), 1 was a case with low anorectal malformation and the other 2 had no other abnormalities. Regarding the comorbidities of the fetus, 10 fetuses had no abnormalities, 1 had isolated mild polyhydramnios, 1 had CF, 1 had isolated mild bilateral pyelectasis, 1 had spondylocostal dysostosis due to Jarcho–Levin syndrome and 4 fetuses had multiple structural malformations, 2 of which had trisomy and 18 with omphalocele. In 8/18 cases, fetal inguinoscrotal hernias were managed with US follow-up. In 1 case, CS was performed at 39 GW the day after diagnosis to prevent bowel strangulation because of bidirectional bowel peristalsis found within the scrotum; however, in this case, no urgent surgery was required at birth, and the baby was operated on at 8 months of life. In the remaining 9/18 cases, antenatal management was not clearly described. No cases of intrauterine fetal death and intrauterine regression were reported. After birth, there was 1 case of spontaneous regression that involved the smallest hernia (2.9 × 1.7 cm) and 4 cases of post-natal death concerning the 4 plurimalformated fetuses. All the other newborns (13/18) required surgical correction of the hernia within 8 months of life, especially during the first month (11/13 cases). In one case, excision of the contralateral torsed testis was also performed because of postnatal evidence of testicular torsion in the opposite side of the hernia. Interestingly, only 2/13 cases required intervention within 72 h from birth, and both of them were fetuses with an absence of blood flow signal during CD assessment of the mass and without intraabdominal bowel dilatation, polyhydramnios and other comorbidities. On the other hand, the only 2/13 cases operated on beyond the first month of life (at 6 and 8 months, respectively) were fetuses with bowel dilatation in the case of anorectal malformation with mild polyhydramnios and no data regarding the CD. The postoperative outcome was uneventful in almost all cases (11/13) since in 2/13 cases, it was not mentioned. However, the case of one of the largest hernias (6.7 × 5 × 5.1 cm) was operated on two times, at 13 and 28 days of life, because of the development of a contralateral inguinoscrotal hernia.

## 5. Discussion

According to our case report, only 19 cases of fetal inguinoscrotal hernia are reported in the current literature. The causative agent underlying the pathogenesis of this condition in utero is still not completely understood. The herniation of the bowel loops into the scrotum may occur together with testicular descent through the processus vaginalis between the seventh and the ninth month of pregnancy. The two main factors involved in the pathogenesis of postnatal inguinoscrotal hernia are the PPV and the increased pressure in the abdominal cavity—for example, after a vigorous cry [4]. However, it is still unclear which factors may lead to increased intraabdominal pressure during fetal life since it is usually similar to intra-amniotic pressure and since it is frequently an isolated disorder. Some intraabdominal disorders may be responsible for the elevation of abdominal pressure, such as all causes of bowel obstruction with consequent bowel dilatation. In the case of Jarcho–Levin Syndrome, the increased intra-abdominal pressure seemed secondary to the short/rigid thorax of the fetus and the rapidly developing abdominal contents with advancing gestation [6]. Moreover, in cases of omphalocele, it has been hypothesized that an intrinsic defect of the abdominal wall is the possible causative agent rather than the increase in intraabdominal pressure [12,13]. The data collected in our paper show that in 68% (13/19) of cases, inguinoscrotal hernia affects the right side, and it is more frequently an isolated finding (10/19). We found only 6/19 cases with associated major structural anomalies/genetic disorders (trisomy 18 with omphalocele, CF, low anorectal malformation with perineal fistula, spondylocostal dysostosis due to Jarcho–Levin syndrome, various left foot deformities) and 3/19 cases with minor defects (mild pyelectasis and/or mild polyhydramnios). Fetal inguinoscrotal hernia typically occurs in the third trimester, and it appears on the US as a fetal scrotal mass with the following ultrasonographic features: mainly solid content (6/19), with smooth scrotal contours, with a nonhomogeneous (10/19) or echogenic echotexture (5/19) and a few small echo-free cystic areas (representative of the bowel loop content) (7/19). The contralateral testis can be found as peripherally displaced (4/19), while the homolateral one can be difficult to be identified (6/19). The blood flow signal of the herniated vessels from the mesentery artery can be sometimes confused with the vascularization of a tumor [4], and its absence can be a sign of obstruction of the herniated bowel. The visualization of movements and deformations of echo-free/cystic components inside the mass is the pathognomonic sonographic feature, as it reveals the intestinal content of the lesion (16/19). However, the absence of bowel peristalsis does not exclude the diagnosis, as in our case; sometimes it can be detected at sonographic re-assessment, and sometimes it can be absent in cases of bowel incarceration. In difficult cases, a fetal MRI complementary to the US can be crucial for diagnosis, as it can reveal the content of the mass. In our case, MRI was not performed since the birth was already scheduled for a few hours after diagnosis due to obstetric reasons. Moreover, a sagittal scan of the fetus may be helpful to detect the herniation of the bowel through the abdominal wall into the scrotum. In addition, perhaps the developmental time of the lesion could also help in the diagnosis, since in our case, the scrotal mass had formed in about 2 weeks, hiding the possibility of the rapid development of the lesion. A differential diagnosis from the other scrotal masses of the fetus essentially consists of five conditions: hydrocele, testicular torsion, testicular teratoma, meconium hydrocele and sacrococcygeal teratoma. Hydrocele is the most common one, and it is due to the passage of peritoneal fluid into the scrotum through the PPV, creating a “half-moon” of anechogenic fluid that surrounds the testis [12]. Testicular torsion is characterized by the increased size of the testis and epididymis, the absence of flow signal on CD and the accumulation of hemorrhagic fluid between the visceral and parietal layers of the tunica vaginalis and outside the tunica vaginalis, giving a typical image of a “double ring hemorrhage” [7,15,19]. Testicular teratoma is the most common testicular tumor but is rarely diagnosed prenatally [15]. Its US pattern is similar to that of the ovarian dermoid cyst, with the absence of testis in the scrotal sac and peristaltic movements [20,21,22]. Meconium hydrocele sonographically appears as a scrotal solid mass (since meconium is echogenic), with calcifications within the scrotum and the associated abdominal signs of meconium peritonitis (ascites, punctiform hyperechogenic lesions around the peritoneal cavity or abdominal pseudocyst) [11]. Sacrococcygeal teratoma is a rare benign tumor that can extend to the scrotum. It usually presents as a mixed-ecostructure mass that is highly vascularized during a CD examination. Compared with inguinoscrotal hernia, it is usually larger and without peristaltic movements [8,12]. Once a diagnosis of fetal inguinoscrotal hernia is made, the fetus should be carefully examined for other structural anomalies associated (especially in the abdominal site) and monitored for signs of intestinal obstruction/strangulation (abdominal bowel dilatation, polyhydramnios and absence of blood flow signal). Attention should also be paid to the contralateral scrotum since it could be the site of hydrocele, hernia and/or testicular torsion. If features of obstruction are present, the delivery should be planned if the grade of obstruction impacts fetal well-being. In fact, the presence of signs of bowel obstructions in the US does not seem to change postnatal morbidity, if they are isolated. In our paper, in fetuses with no abnormalities, the presence of intraabdominal bowel dilatation alone or polyhydramnios alone did not require a different or urgent postnatal treatment and did not change the postoperative outcome. Probably, they are more predictive of bowel strangulation when they appear together. However, the absence of blood flow signal within the scrotal mass at CD examination seems to predict a more severe grade of obstruction, as we have found it to be associated in 2/4 cases with urgent surgical correction within 72 h from birth. After birth, almost all inguinoscrotal hernias require surgical correction since there is a 30–40% risk of incarceration and possible strangulation in the first year of life [23]. Spontaneous postnatal regression is rare and usually occurs in smaller hernias. The limitations of this study are due to the small sample size. More data are needed to confirm and validate the proposed results and considerations.

## 6. Conclusions

Although it is a rare condition in fetal life, inguinoscrotal hernia must always be considered in the differential diagnosis of fetal scrotal mass. It typically presents on the US as a mainly solid mass, more frequently in the right scrotum, with mixed/hyperechoic echotexture and a few small echo-free cystic areas in the interior. The identification of bowel peristalsis of the echo-free cystic areas within the scrotum is a pathognomonic sign but not always easily visible. In case of doubt, a longitudinal scan of the fetal abdomen and/or a fetal MRI can be diriment, as they can define, respectively, the herniation of the bowel through the abdominal wall into the scrotum and the bowel content of the scrotal mass. Moreover, the rapid time of development of the lesion could be helpful in differential diagnosis. Once diagnosed, a longitudinal US follow-up has to be carried on to monitor for signs of bowel obstruction (polyhydramnios, abdominal bowel dilatation and absence of blood flow signal) and eventually decide upon urgent delivery in the case of signs of incarceration affecting fetal well-being, since the prediction of bowel strangulation seems to be poor if polyhydramnios and abdominal bowel dilatation do not appear together and the blood flow signal is present.

## Figures and Tables

**Table 1:** Literature update on fetal inguinoscrotal hernia. (1945–September 2023).

Ref.	Sample Size	N°	GA at Diagnosis (W)	Side	Bowel Peristalsis	Size (cm)	Blood Flow Signal	US Findings	Bowel Abdominal Dilatation	Fetal Comorbidities	MRI Findings	Treatment	Mode of Delivery	GA at Birth (W)	Postnatal Treatment	Post-Operative Outcome
[3]	2	1	39	Right	Present and bidirectional	4.6 × 3.5 × 3	Present	Mass with nonhomogeneous echotexture, predominantly solid, with few small echo-free cystic areas	Present (16 mm)	N	Signal intensity similar to SB that extended from the abdominal cavity into the scrotum	CS the day after for suspicion of intestinal obstruction and incarcerated hernia (bidirectional bowel peristalsis within the scrotum)	Urgent CS (prevention of strangulation)	39	Surgical correction at 8 M	U
		2	36	Right	Present	ND	Present	Enlarged scrotum with an echogenic mass inside	ND	N	Scrotum filled with SB which presented as hyperintense	ND	VD	38	Surgical correction at 1 M	U
[4]	1	3	37	Right	Present	2.9 × 1.7	ND	Heterogeneous echotexture. Displacement of homolateral testis. Irregular contours of the scrotum due to movements of intrascrotal echoes	ND	N	N	ND	VD	39	Spontaneous regression after birth	-
[5]	1	4	31	Right	Present	ND	ND	Evidence of bowel loops within the scrotum	Present (13–17 mm)	CAVB, low anorectal malformation with a perineal fistula, bowel dilatation, mild polyhydramnios	N	US FU	Urgent CS (fetal Doppler abnormalities)	34	Surgical correction at 6 M	ND
[6]	1	5	33	Left	Present	ND	ND	Hyperechogenic scrotal mass. Sagittal view showed herniation of the bowel through the ventral abdominal wall into the scrotum	ND	Spondylocostal dysostosis (Jarcho–Levin syndrome)	N	ND	VD	40	Died on day 3 due to RDS	-
[7]	1	6	35	Right	Present (noted at II examination)	5.5	Present	Heterogeneous scrotal mass containing echogenic areas	Absent	Mild bilateral pyelectasis	N	US FU	VD	40	Surgical correction at 4 D	U
[8]	1	7	21	Right	Present (noted 2 W after I examination)	21 W → 3.3 × 3.0 36 W → 4.1 × 4.7 × 4.8	Absent	Mass with complex echogenicity, predominantly solid with scattered small echo-free/cystic components. Homolateral testis not identified, contralateral testis displaced peripherally	Absent	N	N	US FU	CS (non-reassuring FHR on CTG)	37	Surgical correction after birth	U
[9]	1	8	34	Left	Present	34 W → 3.6 × 3.0 37 W → 4.8 × 3.2	ND	Complex mass with peristalsis; homolateral testis not identified	Present (bowel dilated and filled with echogenic meconium)	Cystic fibrosis	N	US FU	VD	ND	Surgical correction at 3 W	U
[10]	1	9	34	Right	Absent	3.2 × 2.7	Absent	Nonhomogeneous echogenicity, homolateral testis not clearly identified. Contralateral testis displaced peripherally	Absent	N	N	ND	CS (pPROM)	36	Surgical correction at 3 D	U
[11]	1	10	36	Right	Present (noted at II examination performed the day after)	36 W → 4.6 × 5.1 × 5.3 39 W → 6.7 × 5.5 × 5.1	Absent	Homolateral testis not identified; contralateral testis displaced peripherally. Mass with complex echogenicity, predominantly solid with scattered small echo-free components	Present. Single dilated loop of SB measuring 5.5 × 3.0 × 3.2 cm was noted at 39 W within the fetal abdomen	N	N	Weekly US FU	VD	ND	Surgical correction at 13 D	Development of a large reducible left-sided indirect inguinal hernia, which was operated on at 28 D
[12]	1	11	30	Right	Present	4.2	ND	Mass with mixed echostructure, with small fluid filled cystic spaces	ND	IUGR polyhydramnios, Trisomy 18, (omphalocele, clubfeet, clenched hands, large atrioventricular septal defect with a common atrioventricular valve)	N	US follow up (4 W after)	VD	37	Died 3 h after birth	-
[13]	1	12	33	Right	Present	ND	ND	Appearance of right scrotal tumor	Absent	IUGR, polyhydramnios Trisomy 18 (ventricular septal defect, an omphalocele, brachycephaly, bilateral clubfeet and hand abnormalities with crossed fingers, micropenis)	N	ND	VD	35	Died within 1 h	-
[14]	1	13	36	Left	Absent	4.2 × 5 × 3.8	Present	Solid mass, predominantly echogenic without a cyst. Homolateral testis not identified; the contralateral displaced laterally	Absent	Various left foot deformities	Signal intensity similar; SB on both longitudinal and transverse relaxation time-weighted imaging	ND	CS	37	Died at 5 D (multiple joint contractures and failure to thrive)	-
[15]	2	14	36	Bilateral	Present	4.2 × 3.0	Present	Bilateral scrotal masses, visualization of bowel peristalsis	Absent	Mild polyhydramnios	N	ND	VD	38	Operated 1 M	U
		15	24	ND	Present	ND	ND	Swollen, enlarged scrotum with an echogenic mass inside the scrotum	ND	N	N	ND	ND	ND	Operated at 1 M	U
[16]	1	16	37	Right	Present (noted at II examination performed 1 W later)	5.0 × 4.6	Present	Echogenic mass, mixed echostructure and regular walls, containing a few small echo-free cystic areas. Hydrocele in the contralateral side	Absent	N	N	US FU	CS (breech presentation)	38	Operated at 10 D	U
[17]	1	17	39	Right	Present	4	Present	Complex, solid-appearing mass. Normal contralateral testis	ND	N	N	ND	VD	ND	Surgical repair of hernia and excision of contralateral torsed testis in the neonatal period	ND
[18]	1	18	33	Left	Present	6.5 × 5.6 × 6.4	ND	Moving, echo-free, cyst-like structures in an enlarged scrotum	Absent	N	N	US FU (2 W intervals)	VD	40	Operated at 4 D	ND

GA, gestational age; W, weeks; D, days; M, months; H, hours; FU, follow up; N°, case number; CS, cesarean section; VD, vaginal delivery; MRI, magnetic resonance imaging; US, ultrasound; ND, no data available; N, none; U, uneventful; IUGR, intrauterine growth restriction; SB, small bowel; RDS, respiratory distress syndrome; FHR, fetal heart rate; CTG, cardiotocography; CAVB, complete atrioventricular block; pPROM, preterm premature rupture of membranes.

## Data Availability

Data are contained within the article.
